# Unraveling High‐Yield Phase‐Transition Dynamics in Transition Metal Dichalcogenides on Metallic Substrates

**DOI:** 10.1002/advs.201802093

**Published:** 2019-01-25

**Authors:** Xinmao Yin, Chi Sin Tang, Di Wu, Weilong Kong, Changjian Li, Qixing Wang, Liang Cao, Ming Yang, Yung‐Huang Chang, Dianyu Qi, Fangping Ouyang, Stephen J. Pennycook, Yuan Ping Feng, Mark B. H. Breese, Shi Jie Wang, Wenjing Zhang, Andrivo Rusydi, Andrew T. S. Wee

**Affiliations:** ^1^ Department of Physics Faculty of Science National University of Singapore Singapore 117542 Singapore; ^2^ Singapore Synchrotron Light Source (SSLS) National University of Singapore Singapore 117603 Singapore; ^3^ NUS Graduate School for Integrative Sciences and Engineering National University of Singapore Singapore 117456 Singapore; ^4^ International Collaborative Laboratory of 2D Materials for Optoelectronics Science and Technology Shenzhen University Shenzhen 518060 China; ^5^ Hunan Key Laboratory of Super‐microstructure and Ultrafast Process School of Physics and Electronics Central South University No. 932, South Lushan Road Changsha Hunan Province 410083 China; ^6^ Department of Materials Science and Engineering National University of Singapore 9 Engineering Drive 1 Singapore 117575 Singapore; ^7^ Anhui Province Key Laboratory of Condensed Matter Physics at Extreme Conditions High Magnetic Field Laboratory of the Chinese Academy of Sciences Hefei 230031 China; ^8^ Institute of Materials Research and Engineering A∗STAR (Agency for Science, Technology and Research) 2 Fusionopolis Way Singapore 138634 Singapore; ^9^ Bachelor Program in Interdisciplinary Studies National Yunlin University of Science and Technology Yunlin 640 Taiwan

**Keywords:** high‐resolution transmission electron microscopy, high‐yield phase transition for transition metal dichalcogenides, interfacial hybridizations, inverted gap, optical spectroscopy

## Abstract

2D transition metal dichalcogenides (2D‐TMDs) and their unique polymorphic features such as the semiconducting 1H and quasi‐metallic 1T′ phases exhibit intriguing optical and electronic properties, which can be used in novel electronic and photonic device applications. With the favorable quasi‐metallic nature of 1T′‐phase 2D‐TMDs, the 1H‐to‐1T′ phase engineering processes are an immensely vital discipline exploited for novel device applications. Here, a high‐yield 1H‐to‐1T′ phase transition of monolayer‐MoS_2_ on Cu and monolayer‐WSe_2_ on Au via an annealing‐based process is reported. A comprehensive experimental and first‐principles study is performed to unravel the underlying mechanism and derive the general trends for the high‐yield phase transition process of 2D‐TMDs on metallic substrates. While each 2D‐TMD possesses different intrinsic 1H‐1T′ energy barriers, the option of metallic substrates with higher chemical reactivity plays a significantly pivotal role in enhancing the 1H‐1T′ phase transition yield. The yield increase is achieved via the enhancement of the interfacial hybridizations by the means of increased interfacial binding energy, larger charge transfer, shorter interfacial spacing, and weaker bond strength. Fundamentally, this study opens up the field of 2D‐TMD/metal‐like systems to further scientific investigation and research, thereby creating new possibilities for 2D‐TMDs‐based device applications.

## 1 Introduction

Low‐dimensional materials hold immense promise as the primary building blocks in creating novel architectures for electronic and optoelectronic applications. In particular, 2D transition metal dichalcogenides (2D‐TMDs) with MX_2_ stoichiometry, where M is a transition metal and X is a chalcogen, have attracted much attention due to their potential applications in the fields of spintronics,^1^ valleytronics,^2^, ^3^ optoelectronics,^4^ and field‐effect transistors (FETs).^5^ 2D‐TMDs can manifest in different polymorphic phases and physical properties as revealed by several experimental and theoretical studies.^6^, ^7^ For example, the semiconducting 1H‐phase is stable at ambient conditions for Group VI TMDs such as MoS_2_ and WSe_2_, which is important for the aforementioned applications. In addition, there exists the metallic 1T‐phase which will spontaneously relax to a distorted 1T′ quasi‐metallic phase with a fundamental gap and an inverted gap.^7^, ^8^ The 1T′‐phase 2D‐TMD has a potential range of applications such as supercapacitor electrodes,^9^ hydrogen evolution reaction catalysts,^10^, ^11^ dipolar ferroelectricity,^12^ charge density wave formation,^13^ and Weyl semimetal.^14^, ^15^, ^16^, ^17^, ^18^

Given the immense promise that each TMD phase holds, their phase transition processes is an important field of research. Experimental techniques that induce such processes include alkali metal intercalation,^19^ electron beam irradiation,^20^ mechanical strain, electron donation,^21^ and plasmonic hot electrons.^22^ However, the yield of 1T′‐phase 2D‐TMDs is found to be low due to air exposure^23^ and effects of ageing.^24^ While the limitation may hamper the potential development of device applications based on 2D materials, the ability to increase the phase‐transition yield can significantly facilitate the scaling of such novel materials for wide‐scale applications. Our previous study reported a straightforward and convenient way of inducing the 1H‐to‐1T′ phase transition in a specific system, monolayer‐MoS_2_ on Au substrate, based on an annealing process.^25^ Even though this simple process holds immense promise in the development of device applications such as the fabrication of 2D‐TMD‐based field‐effect transistors, the optimum percentage yield of 1T′‐phase MoS_2_ is only in the range of ≈37%. Hence, an effective high‐yield method to obtain large‐area 1T′‐phase 2D‐TMDs remains a key challenge for wide‐scale device applications. More importantly, unraveling the inherent charge dynamics and physical mechanisms underlying this phase transition behavior is of fundamental importance. Detailed mechanistic studies will help to improve the phase transition efficiency.

In this work, we report a comprehensive study using high‐resolution transmission electron microscopy (HRTEM), spectroscopic ellipsometry, transport, Raman, photoluminescence (PL), synchrotron‐based photoemission spectroscopies (PES), and first‐principles calculation, of the 1H‐1T′ structural transformation of 2D‐TMDs on different metallic substrates. We demonstrate that interfacial hybridizations between 2D‐TMDs and metallic substrates can be enhanced via high‐temperature annealing, which is the key mechanism resulting in the 1H‐1T′ phase transition of the 2D‐TMDs on metallic substrates. A computation study involving a series of metallic substrates (graphene, Au, Ag, Cu) demonstrates stronger interfacial hybridizations in the MoS_2_/Cu system as compared to MoS_2_/Ag, MoS_2_/Au, and MoS_2_/graphene systems. To verify our computational predictions, we performed annealing‐based experimental investigations and achieved an ≈85.7% yield of the 1T′‐phase MoS_2_ at an optimum annealing temperature of 300 °C in the MoS_2_/Cu system, which is significantly higher than the ≈37% yield of 1T′‐phase MoS_2_/Au previously reported.^25^ On a different 2D‐TMD, an ≈58.8% yield of 1T′‐phase WSe_2_ is achieved for monolayer‐WSe_2_ on gold (WSe_2_/Au) upon annealing at 250 °C. The ability to significantly increase the 1H‐1T′ phase transition yield greatly facilitates the scaling of 2D‐TMDs for wide‐scale applications. The unprecedented quality of the 2D‐TMD/metallic interface is demonstrated at the atomic length scale. Moreover, we experimentally observe the tunable inverted mid‐gap in 1T′‐phase WSe_2_. This experimental observation shows the pivotal role that electron–electron correlation plays in the electronic structure of 2D‐TMDs, and its effects on their energy bandgap. It allows us to better identify and manipulate novel materials with intriguing many‐body effects for use in numerous nanoscale optoelectronic applications.

Therefore, this comprehensive study further advances the prospect of phase engineering of 2D‐TMDs and it serves as an ideal reference for material scientists for the controlled growth of 2D‐TMDs on metallic substrates. Sample details and detailed experimental descriptions are explained in the Experimental Section.

## 2 Results and Discussion

### 2.1 Substrate‐Dependent First‐Principles Study

**Figure 1:**
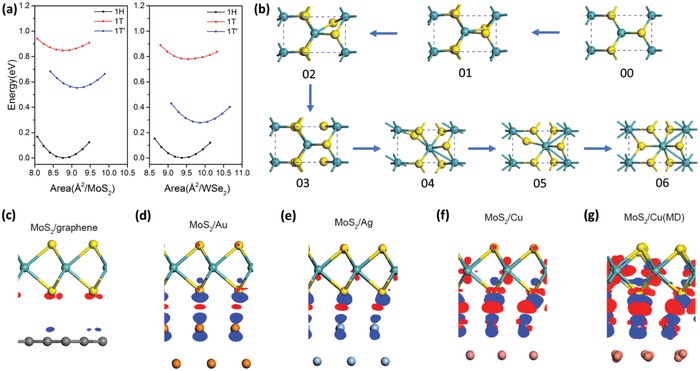
Phase transition dynamical simulation for MoS_2_ on metallic substrates. a) Equation of states of MoS_2_ and WSe_2_ in 1H, 1T, and 1T'‐phase, respectively. The energy minimum in the curve of 1H‐phase was used as the reference. b) The structural evolution of 1T′‐phase (06) monolayer‐MoS_2_ from 1H‐phase (00) through the intermediate phases. Blue spheres denote the W atoms while the yellow are S atoms. c–f) The visualized charge density difference of 1H‐MoS_2_ on graphene, Au, Ag, and Cu substrates, where the isosurface values for MoS_2_/Au, MoS_2_/Ag, and MoS_2_/Cu are 8.0 × 10^−4^ e Å^−3^, while it is 1/10 for MoS_2_/graphene. The red dots denote the charge accumulation region while the blue dots denote the charge depletion regions. (See also Figure S6, Supporting Information.) g) The visualized charge difference of monolayer 1H‐MoS_2_/Cu substrates by using an isosurface value of 8.0 × 10^−4^ e Å^−3^ after the molecular dynamics simulations (MoS_2_/Cu (MD)) with the annealing temperature of 550 K.

First‐principles calculation result indicates that the energy of the distorted 1T′ quasi‐metallic phase in equilibrium state is lower than that of the metallic 1T‐phase with rhombohedral stacking for both monolayer MoS_2_ and WSe_2_, as shown in **Figure** **1**a. This suggests that both MoS_2_ and WSe_2_ in 1T′‐phase are more stable than their 1T‐phase counterparts. It also shows that monolayer MoS_2_ and WSe_2_ are both 1H‐phase in their ground state. To study the structural phase transition dynamics of the 2D‐TMD system, first‐principles calculations are conducted to simulate the 1H‐1T′ transition in freestanding monolayer‐MoS_2_ (details in the Experimental Section). The schematic of climbing images using the nudged elastic band (c‐NEB) method in Figure 1b identifies the distinct intermediate phases between the initial 1H (Step 00) and the final 1T′‐MoS_2_ (Step 06) structure, while other images display the continuous changes of the structure. This 1H‐1T′ structural transition involves the edge sulfur atoms moving to the center and the central sulfur atoms sliding to the edge along the zig‐zag direction, while the central Mo atom slides slightly along the armchair direction. To examine how the choice of substrate affects the interfacial interactions that mediate the 1H‐1T′ phase transition, further computation study is conducted to compare the interfacial dynamics between 1H‐phase MoS_2_ on graphene, Au, Ag, and Cu substrates. Results unveil significant differences in the interfacial hybridizations between monolayer‐MoS_2_ and different substrates through the representation of interfacial charge transfer, distance, and binding energy (compiled parameters in **Table** **1** where the binding energy is defined as the energy difference between the heterostructure and its individual components). By comparing the results of these interfacial parameters, notice that there is an appreciably stronger interfacial hybridization between the 1H‐phase monolayer‐MoS_2_ and Cu substrate as compared to the other substrates (Cu > Ag > Au > graphene). Substrate‐to‐monolayer charge transfer correspondingly shows an increasing trend from MoS_2_/graphene→MoS_2_/Au→MoS_2_/Ag→MoS_2_/Cu as visualized in Figure 1c–f. The charge accumulation on the Mo atomic layers and charge depletion between Mo and interfacial S atomic layers indicate the weakened Mo—S bond strength, thus, decreasing the 1H‐1T′ phase transition barrier (detail in the Experimental Section). This in turn greatly facilitates the phase transition process. The collective trend of our findings shows that interfacial hybridizations of TMD/substrate systems increases with increasing chemical reactivity of the metallic substrates. Graphene, a weakly interacting 2D electrode, has the weakest interfacial hybridizations with 2D‐TMD due to the inert graphene surface. As shown experimentally later, the 1H‐1T′ phase transition yield of 2D‐TMD is directly influenced by this variation in interfacial hybridizations of the TMD/substrate systems.

**Table 1:** Calculated interfacial hybridizations: binding energy, charge transfer, and interfacial distance of different heterostructures, between MoS_2_ and metallic substrates. The binding energy is the energy difference of the heterostructure and its individual components. All charge transfers are from the substrate to 1H‐MoS_2_. The interfacial distance is defined as the distance between the highest atomic layer of substrate and the lowest atomic layer of MoS_2_

	Binding energy [meV Å^−2^]	Charge transfer [e^−^ f.u.^−1^]	Interfacial distance [Å]
MoS_2_/graphene (4 × 4/3$\sqrt{3}$ × 3$\sqrt{3}$)a)	−20.5	0.005	3.38
MoS_2_/Au (8 × 8/9 × 9)	−55.7	0.017	2.68
MoS_2_/Ag (8 × 8/9 × 9)	−62.5	0.071	2.50
MoS_2_/Cu (4 × 4/5 × 5)	−90.8	0.126	2.16
MoS_2_/Cu(MD) (4 × 4/5 × 5)	−93.3	0.154	1.78

^a)^ The in‐plane size of MoS_2_ or substrate supercell used to compose the heterostructure. MoS_2_/Cu (MD) denotes the MoS_2_/Cu after molecular dynamics simulations to imitate the high‐temperature annealing process.

Having established the understanding of the interfacial hybridizations between 1H‐phase monolayer‐MoS_2_ and the different substrates, we provide further density functional theory (DFT) studies to examine the effects of the thermal annealing process on the structural and electronic properties of 1H‐MoS_2_ on Cu substrate. Ab initio molecular dynamics simulations were performed to simulate the annealing process of the MoS_2_/Cu system at 550 K. Figure 1g shows the atomistic structure superimposed with the visualized differential charge density for 1H‐MoS_2_/Cu during the simulated annealing process. Simulation results show further enhancement in interfacial hybridizations as compared to the pristine MoS_2_/Cu system (Table 1) evidenced in the further increase in interfacial binding energy (see also Figure S3a, Supporting Information), larger charge transfer (see also Figure S3b, Supporting Information), shorter interfacial spacing (see also Figure S4, Supporting Information), and weaker bond strength (Figure S5, Supporting Information). Therefore, we deduce that the increase in interfacial hybridizations at the TMD‐substrate interface induced by the high‐temperature annealing process is the key underlying mechanism leading to the 1H‐1T′ phase transition of 2D‐TMDs.

### 2.2 Annealing‐Induced Phase Transition of MoS_2_ on Cu

**Figure 2:**
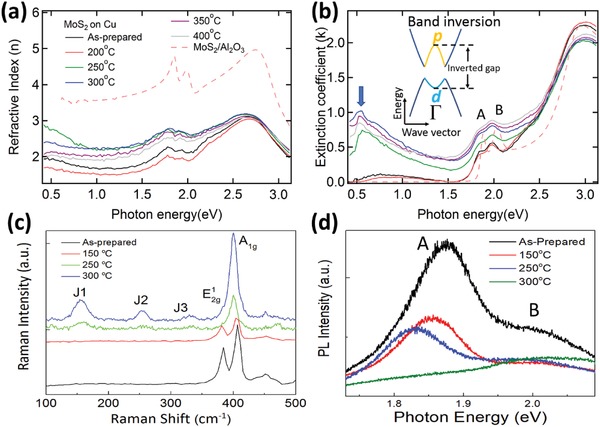
Optical characterization of MoS_2_/Cu at various annealing temperatures. a) Refractive index, *n*(ω), and b) extinction coefficient, *k*(ω), of MoS_2_/Cu annealed at various temperatures and MoS_2_/Al_2_O_3_ measured by high‐resolution spectroscopic ellipsometry. The inset is the schematic band structure of 1T′‐MoS_2_. The lattice distortion along with strong electron–electron correlations in 1T′‐MoS_2_ results in a band inversion around Γ‐point, which lowers the W d‐orbital below the Se p‐orbital and opens an inverted gap. c) Comparing Raman spectra of MoS_2_/Cu before and after annealing at 150, 250, and 300 °C. d) Annealing temperature dependent of photoluminescence spectroscopic results.

The 1H‐1T′ phase transition yield for MoS_2_/graphene is very low at elevated annealing temperatures as reported previously.^26^ In view of the predictions by our computational study on the trend of the high‐yield phase transition of 2D‐TMDs on metallic substrates, Cu is used as the metallic substrate in our annealing‐based phase transition experimental study in an attempt to achieve a higher yield of 1T′‐phase monolayer‐MoS_2_. This phase transition process of MoS_2_/Cu is monitored using spectroscopic ellipsometry, transport, synchrotron‐based high‐resolution photoemission, Raman, and photoluminescence spectroscopic methods. The distinctive feature differentiating the electronic structure of 1T' from 1H phase 2D‐TMDs is the presence of the inverted gap below the bandgap in the former.^8^ Therefore, we perform spectroscopic ellipsometry measurements of monolayer‐MoS_2_ on Cu to directly observe the inverted gap. **Figure** **2**a,b shows the complex refractive indices (*N*(ω) = *n*(ω) +*ik*(ω)) of MoS_2_ on Cu and Al_2_O_3_ substrates at various annealing temperatures as measured by spectroscopic ellipsometry.

For the refractive index, *n*(ω) in Figure 2a, when MoS_2_ is transferred from Al_2_O_3_ onto Cu, the intensity decreases significantly. This may be due to the increased electron‐screening effect from the Cu substrate. After annealing, *n*(ω) increases in the spectral region between 0.4 and 2.5 eV when the sample is annealed at 300 °C, it then reduces upon further annealing at 350 °C and above.

The extinction coefficient spectra, *k*(ω) in Figure 2b, show several strong absorption peaks attributed to optical band transitions. Excitonic absorption peaks A and B arise due to direct gap transitions at the K/K′ points in the Brillouin zone of MoS_2_. In comparison with monolayer‐MoS_2_/Al_2_O_3_ (dashed line), peaks A and B of MoS_2_/Cu show a redshift, attributed to charge transfer from copper to MoS_2_ 27 and electron‐screening effect in copper which suppresses the Wannier excitons in MoS_2_.

Interestingly, the *k*(ω) show a broad mid‐infrared peak below excitonic peak A. Note that there are no mid‐infrared peaks in the 1H‐structure MoS_2_ (Figure 2b, MoS_2_/Al_2_O_3_). At the annealing temperature of 300 °C, the intensity of the mid‐infrared peak is maximized while the threshold energy of this peak is redshifted. The energy position of the mid‐infrared peak (≈0.5 eV) is close to the calculated value of the inverted gap of 1T′‐MoS_2_.^8^ A similar mid‐infrared peak has also been observed in 1T′‐MoS_2_ on Au and has been ascribed to the inverted gap.^25^ This inverted gap, a signature of 1T′‐phase 2D‐TMD, is located at the Γ‐point in the 2D Brillouin zone.^8^

We seek to rule out other possible causes of the mid‐infrared optical peak at ≈0.5 eV such as vacancies introduced during sample growth, and the presence of physisorbed impurities introduced during sample transfer. While the presence of intrinsic vacancies in chemical vapor deposition (CVD)‐grown monolayer‐MoS_2_ has been reported,^28^, ^29^ it can be ruled out as the cause of the ≈0.5 eV optical peak because it only appears after the transfer of MoS_2_ onto Cu and is absent in MoS_2_/Al_2_O_3_.^30^ Second, there is an increase in the optical peak intensity at ≈0.5 eV with rising annealing temperature. However, sample annealing in ultrahigh vacuum should result in the desorption of absorbed impurities. Hence, we can also rule out the possibility of physisorbed impurities being introduced during the transfer process as a contributing factor to this ≈0.5 eV mid‐infrared peak.

Raman spectroscopy is also an effective method to characterize 1T′‐phase structures in 2D‐TMDs via the analysis of the Raman modes. A high‐resolution Raman study is conducted to support the 1H‐1T′ phase transition hypothesis in the monolayer‐MoS_2_/Cu system with the spectra displayed in Figure 2c. The spectrum of the as‐prepared sample shows two main modes—the in‐plane $E_{2}^{1}g$ mode (opposing vibrations of the in‐plane Mo and S atoms) and the out‐of‐plane A_1g_ mode (opposing vibrations of the two out‐of‐plane S atoms). After annealing the sample up to 300 °C, the intensity of the $E_{2}^{1}g$ peak reduces significantly. In contrast, the A_1g_ peak remains robust amid the phase‐transition process. This result is similar to other high‐yield 1T′‐phase Raman studies.^31^, ^32^ This observation in the Raman spectra is due to the localized phase‐transition process and that the Raman mode intensity depends mainly on the electron–phonon coupling strength in the Raman processes.^33^ Most importantly, the Raman spectrum of MoS_2_/Cu after annealing at 300 °C shows three prominent features at ≈154 (J1), ≈248 (J2), and ≈322 cm^−1^ (J3) which are absent from the as‐prepared state. These are characteristic peaks of the 1T′‐octahedral structure^22^, ^25^, ^34^ and are consistent with previous studies where the 1T′‐phase MoS_2_ have been observed.^25^, ^34^

High‐resolution photoluminescence measurements are also conducted on the monolayer‐MoS_2_/Cu sample before and after annealing. The spectral changes are displayed in Figure 2d. For the as‐prepared sample, exciton peaks A and B located at ≈1.89 and ≈2.03 eV, respectively, are attributed to the direct bandgap photoluminescence from the K‐point.^35^ Their energy positions are consistent with other monolayer‐MoS_2_ studies and this indicates that the monolayer‐MoS_2_/Cu sample is of high quality. By annealing the monolayer‐MoS_2_/Cu sample at elevated temperatures, peak A is redshifted, broadened and reduced in intensity. Peak A eventually disappears upon further annealing at 300 °C. This is opposite to the effect of a previous 1T′‐1H phase transition study.^19^ Our previous study of the monolayer‐MoS_2_/Au system^25^ also confirms similar PL changes observed as indicative of the transformation of monolayer‐MoS_2_ from the semiconducting 1H to metallic 1T′‐phase.

**Figure 3:**
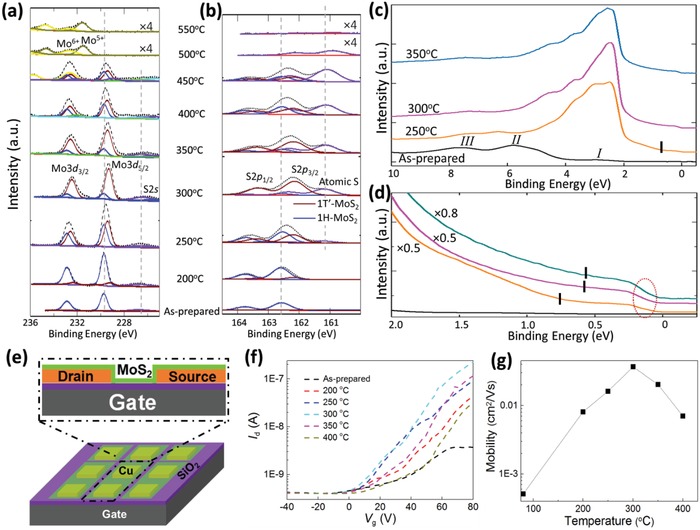
Experimental characterization of MoS_2_ on Cu at various annealing temperatures. a,b) Synchrotron‐based X‐ray photoemission spectra of Mo3d, S2s, and S2p core‐level peak regions for MoS_2_ on Cu film annealed at various temperatures. These spectra can be well fitted by two components with constant peak positions and full width at half maximum. c) Valence band and d) narrow valence band for MoS_2_ on Cu film annealed at various temperatures. The Fermi surface feature is indicated by the dashed red circle while the mid‐gap feature positions denoted by the black vertical markings (see the Experimental Section for details). e) Device structure schematic of a MoS_2_ field‐effect transistor. f) *I*_d_–*V*_g_ of the MoS_2_ device as functions of annealing temperature. g) Mobility versus temperature at different gate voltages of the device as functions of annealing temperature.

**Figure** **3**a,b shows the evolution of Mo3d, S2s, and S2p core‐level spectra for MoS_2_/Cu before and after annealing at elevated temperatures. The Mo core‐level spectrum of the pristine MoS_2_/Cu shows a doublet feature (Figure 3a) that comprises the Mo^4+^ 3d_5/2_ (≈229.7 eV) and 3d_3/2_ (≈232.9 eV) spin–orbit peaks^36^ and a broad peak at lower binding energy (≈226.5 eV) attributed to the S2s state.^37^ The Mo3d doublet has previously been attributed to the MoS_2_ doublet in 1H‐phase.^19^, ^36^ In Figure 3b, the S core‐level doublet for pristine MoS_2_ is located at ≈162.5 eV (S2p_3/2_) and ≈163.7 eV (S2p_1/2_), respectively. These binding energy positions are consistent with those of S core‐level MoS_2_ in the 1H‐phase. This confirms the presence of pristine 1H‐phase monolayer‐MoS_2_/Cu before annealing.^25^, ^37^

Upon annealing the monolayer‐MoS_2_/Cu sample at elevated temperatures of up to 300 °C, the spectral profiles show a clear redshift in both the Mo3d and S2p core‐level spectra (Figure 3a,b). A broad lower binding energy shoulder of S2p_3/2_ at ≈161.1 eV in Figure 3b is attributed to the presence of elemental S which is due to a partial thermal decomposition of MoS_2_ (consistent with previous study^25^). This indicates chemisorbed S‐atoms on the Cu surface. After annealing the sample at higher temperatures, Figure 3a,b shows that the doublets of both the Mo3d and S2p core‐levels are shifted to higher binding energy. Note that the positions of the reference S2s peak (Figure 3a) and the S2p doublet (Figure 3b) remain unchanged at different annealing temperatures which affirm that PES measured energy is properly calibrated throughout the measurement process. Therefore, these are clear indications that the shift to lower binding energy of the Mo3d and S2p 1H‐doublets at elevated annealing temperatures are signatures of phase transformation. The presence of this new phase prevails until 450 °C. After annealing the sample at even higher temperatures, the monolayer‐MoS_2_ sample decomposes as indicated with the sharp decrease in Mo3d and S2p peak intensities which eventually vanish at 550 °C.

To resolve the features into their respective components, careful peak fittings of two‐component phases were conducted for both the Mo3d and S2p core‐level spectra for MoS_2_/Cu. The relative intensities of each component are extracted and displayed in Table S1 (Supporting Information) for the proportion of the new Mo3d_5/2_ peak which represents the percentage yield in 1T′‐phase MoS_2_ via the annealing process. The proportion of the new phase increases after annealing at 250 °C and maximizes at 300 °C. This is followed by a rapid decrease of both components at even higher annealing temperatures.

After annealing the monolayer‐MoS_2_/Cu sample at 450 °C, the Mo3d doublet appears at a higher binding energy of ≈232.6 eV. This corresponds to the presence of MoO_3_.^25^, ^38^ As the annealing temperature increases further, the Mo3d and S2s peaks associated with the 1H‐phase MoS_2_ disappear while the Mo^5+^ oxidation state starts to dominate. This indicates that the monolayer‐MoS_2_ sample has decomposed completely while MoO*_x_* is formed on the Cu foil surface.^38^ These results show that the Mo atom preferentially bonds with residual oxygen atoms on the Cu substrate surface at annealing temperatures above 450 °C, whereas S atom tends to adsorb on the Cu foil surface at first and thereafter desorbs completely at 550 °C.

The fitting results indicate that the positions of 1H‐MoS_2_ peaks are located at ≈229.7 eV for Mo3d_5/2_ and ≈162.5 eV for S2p_3/2_. They remained fixed in their respective positions when the sample is annealed at elevated temperatures. The new components at ≈229.4 eV (Mo3d_5/2_) and ≈162.2 eV (S2p_3/2_) are attributed to the formation of 1T′‐phase monolayer‐MoS_2_ as a result of the annealing‐induced phase transition—in good agreement with previous reports where the binding energies of 1T′‐phase are lower than those of 1H‐phase.^19^, ^25^

Figure 3c compares the valence band spectra up to 10 eV for monolayer‐MoS_2_/Cu sample before and after it is annealed at 250, 300, and 350 °C. For the as‐prepared sample, the valence band spectra are mainly the result of hybridization between the Mo4d and S3p orbitals^39^ with the observation of three main peaks: I, II, and III. According to previous photoemission spectroscopy studies,^39^, ^40^ Peak I is attributed to the nonbonding and lower‐bonding Mo4d valence bands, peak II can be ascribed to Mo3d hybridized with S3p bands, and peak III is mainly attributed to the low‐energy bonding bands of the S3p orbitals at the M symmetry point.

The intensity of peak I increases significantly and newly formed features appear near the Fermi energy after the MoS_2_/Cu sample is annealed at elevated temperatures. By applying the least‐squares fitting technique for the valence band maximum (VBM) spectrum of the as‐prepared MoS_2_/Cu sample, the apparent VBM location (VBM*) is estimated to be at ≈0.29 eV. The VBM* shifts toward the Fermi level and a Fermi surface feature (Figure 3d) can be observed in the MoS_2_/Cu sample after high‐temperature annealing—consistent with the PES core‐level results where the quasi‐metallic 1T′‐phase MoS_2_ starts forming at these annealing temperatures. Further details can be seen in Figure 3d which displays the narrow valence band spectra of the MoS_2_/Cu sample before and after the corresponding annealing‐based treatment. Note that a mid‐gap feature appears at ≈0.7 eV below peak I (black markings in Figure 3d serve as visual guides, details in the Experimental Section) when the sample is annealed at 250 °C and it is shifted to lower binding energy after annealing at the optimum temperature range of 300–350 °C. This mid‐gap feature is attributed to the band inversion of the Mo3d orbital in 1T′‐MoS_2_ due to the symmetry breaking during phase transition.^8^ Meanwhile, the shifting trend of the energy position of the mid‐gap feature is consistent with that of the inverted gap in the previously reported optical study.^25^ The detection of this mid‐gap feature supports the presence of the 1T′‐phase MoS_2_. The Fermi surface (denoted by red dashed circle in Figure 3d) feature can be clearly seen after the 1H‐1T′ phase transition in the narrow valence band spectra.

A new set of MoS_2_/Cu devices was also fabricated (Figure 3e) to study the transport properties as functions of annealing temperature as shown in Figure 3f,g. Results show that the transport properties of the device are very sensitive to the annealing temperature. The field‐effect mobility is ≈5.1 × 10^−4^ cm^2^ V^−1^ s^−1^ for the as‐prepared device. Note that the mobility of our device is lower as compared to the other mechanically exfoliated FET in literature.^5^ This is a result of two main factors. First, defects introduced during the CVD growth and transfer processes along with other factors such as the inhomogeneity of the sample. Next, different metal electrodes result in different electrical performances due to the Schottky barrier between the MoS_2_ monolayer and metal substrate. The Cu electrodes in MoS_2_/Cu are easily oxidized and this reduces the conductivity of the device. By annealing the sample at elevated temperatures of 200, 250, and 300 °C, the mobility rises significantly to 8.1 × 10^−3^, 0.016, and 0.037 cm^2^ V^−1^ s^−1^, respectively. At even higher annealing temperatures of 350 and 400 °C, the mobility drops to 0.20 and 7 × 10^−3^ cm^2^ V^−1^ s^−1^, respectively. This rising trend in electron mobility indicates that the device performance is optimized and that there is a reduction in device contact resistance upon annealing the sample up to an optimum temperature of 300 °C.

Collectively, the experimental results are clear indications of not just an intimate interaction between the MoS_2_ monolayer and the Cu substrate, but also verify the 1H‐1T′ phase transition of the monolayer‐MoS_2_/Cu after the annealing process.

### 2.3 1H‐1T′ Phase Transition of WSe_2_ on Au

We next investigate whether the 1H‐1T′ phase transition is relevant to other 2D‐TMD materials. WSe_2_, a TMD with a lower 1H‐1T′ phase transition energy barrier, should better facilitate the onset of such an annealing‐based phase transition process.

**Figure 4:**
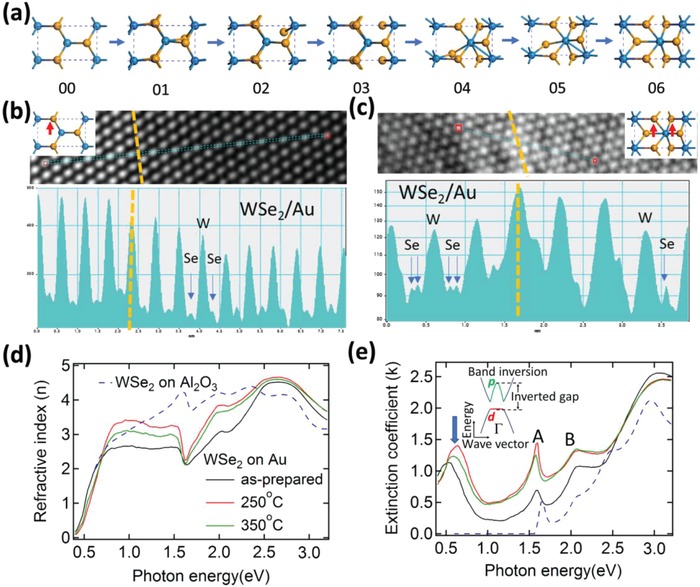
HRTEM and spectroscopic ellipsometry measurements of WSe_2_/Au at various annealing temperatures. a) Structural evolution of 1T′‐phase (06) monolayer‐WSe_2_ from 1H‐phase (00) through the intermediate phases. Blue spheres represent the W atoms while the yellow the S atoms. b,c) Intensity profiles along the blue lines indicated in above HRTEM images of as‐prepared and annealed (at 250 °C) samples. The dark region shows the region where monolayer‐WSe_2_ lies above the Au substrate (WSe_2_/Au), while the other is the region where monolayer‐WSe_2_ is suspended in vacuum. The yellow lines show the boundary between these two regions. d) Refractive index, *n*(ω), and e) extinction coefficient, *k*(ω), of WSe_2_/Au annealed at various temperatures and WSe_2_/Al_2_O_3_ as measured by high‐resolution spectroscopic ellipsometry. The inset shows the schematic band structure of 1T′‐WSe_2_. The lattice distortion together with the strong electron–electron correlations in 1T′‐WSe_2_ cause a band inversion around Γ, which lowers the W d‐orbital below the Se p‐orbital and opens an inverted gap.

Further computational study is conducted to investigate the structural phase transition of freestanding monolayer‐WSe_2_ where **Figure** **4**a displays the NEB schematic of the 1H‐1T′ phase transition process. It is similar to the transition dynamics of the aforementioned monolayer‐MoS_2_.

We perform HRTEM measurement of WSe_2_/Au before and after annealing process at 250 °C. During the HRTEM characterization of the WSe_2_/Au sample, the electrons are strongly absorbed and blocked by the noble metal substrate. Hence, Au thickness has been reduced to 5–10 nm. For a thin Au film (5–10 nm), cavities are inevitably present within the regions of the film. To effectively distinguish the onset of phase transition brought about by the annealing process of WSe_2_/Au, HRTEM characterizations are intentionally performed near the boundaries where monolayer‐WSe_2_ is suspended in vacuum (cavity) and where WSe_2_ lies on top of the Au substrate. The HRTEM images before and after annealing are shown in Figure S9 (Supporting Information). The HRTEM images show clear W lattice for both the as‐prepared and annealed samples, but the Se atoms are not clearly visible. Nevertheless, the distinct structural difference between metallic and semiconducting phases is the movement of Se atoms. The coordination of W with Se in 1H‐WSe_2_ (state 00 in Figure 4a) is trigonal prismatic and the Se atoms in the upper layer are located directly above those in the lower layer. Conversely, the stacking sequence of 1T′‐WSe_2_ is such that the Se atoms in the upper and lower planes are offset from each other (state 06 in Figure 4a). The 1H‐to‐1T′ structural transformation takes place by the gliding one of the Se planes. Therefore, we use the intensity profile to identify the Se intensity variations across the Au boundary, which is normally used to distinguish the metallic phase.^7^, ^41^ Figure 4b,c shows the intensity profiles along the blue lines indicated in the HRTEM images of the as‐prepared and annealed samples, respectively. The yellow lines show the boundary of WSe_2_ on Au and WSe_2_ suspended in vacuum. In the as‐prepared sample (Figure 4b), both the monolayer‐WSe_2_ on Au and WSe_2_ suspended in vacuum show the 1H atomic configuration. Se atoms in the upper layer are located directly above those on the lower layer. Therefore, there is only one Se atomic peak between two W atomic peaks.

After annealing (Figure 4c and Figure S10, Supporting Information, are in different areas of the same sample), for WSe_2_ on Au region, two Se atomic peaks between two W atomic peaks are observed for the WSe_2_ on Au regions. This is due to the offsetting Se atoms between the upper and lower planes in the metallic phase of monolayer‐WSe_2_. The results are consistent with the previous TEM studies of metallic phase 2D‐TMDs.^7^, ^41^ It is noteworthy that there is no obvious zigzag configuration in the HRTEM image (Figure S9, Supporting Information) due to two main reasons. First, Au substrate of thickness 5–10 nm is used to reduce the absorption in electron intensity. As a result, the Au substrate is not sufficiently thick to result in the large‐area 1H‐1T′ phase transition. In addition, the distorted zigzag W atomic chains are unstable under electron beam and they can be relaxed during the imaging process. Similar issue has also been observed in previous TEM studies.^7^, ^41^

To directly observe the inverted gap of 1T′‐WSe_2_, high‐resolution spectroscopic ellipsometry measurements are performed. Figure 4d,e shows the complex refractive indices (*N*(ω) = *n*(ω) + *ik*(ω)) of WSe_2_ on Au at various annealing temperatures and Al_2_O_3_ as measured by spectroscopic ellipsometry. The refractive index, *n*(ω), increases significantly in the spectral region of 0.8–2.5 eV when the sample is annealed at 250 °C (Figure 4d). It then reduces upon further annealing at 350 °C.

The extinction coefficient spectra, *k*(ω), in Figure 4e show several strong absorption peaks, attributed to optical band transitions. The excitonic absorption peaks A and B, arising from direct gap transitions at the K/K′ points in the Brillouin zone of WSe_2_, are identified at ≈1.60 and ≈2.06 eV, respectively. When WSe_2_ is transferred from Al_2_O_3_ onto Au substrate, while both peaks register an increase in their intensities, the excitonic peak B remains in its position and excitonic peak A is redshifted. The redshift of the excitonic peak A is due to the onset of electron transfer from gold to WSe_2_ monolayer.^27^

Similar to the MoS_2_/Cu system after annealing, the extinction coefficient spectra (Figure 4e) show a broad mid‐infrared peak below the excitonic peak A (indicated by the blue arrows in Figure 4e) and its intensity is maximized at the annealing temperature of 250 °C. This is absent from WSe_2_/Al_2_O_3_. The energy position of the mid‐infrared peak (≈0.66 eV) is similar to the calculated value of the inverted gap of 1T′‐WSe_2_.^8^ Therefore, the collective detection of this inverted gap via the means of high‐resolution PES and spectroscopic ellipsometry affirms the presence of the monolayer‐WSe_2_ 1T′‐phase after the high‐temperature annealing process.

Note that the mid‐infrared peak intensity increases alongside the spectral weight in the spectral region below 2.5 eV after annealing the sample. Meanwhile, there is a decrease in spectral weight in the energy region above 2.5 eV, and this indicates a spectral‐weight transfer to the energy region below 2.5 eV. The large energy range spectral‐weight transfer after annealing (from high to low‐energy region) reveals that electron–electron correlations play a crucial role in the origin of the mid‐infrared peak. It also indicates the presence of strong electronic correlations in the WSe_2_/Au system, which unlike freestanding WSe_2_, directly affect the energy bandgap of this material. The presence of the mid‐infrared peak in the as‐prepared WSe_2_/Au sample (80 °C treatment) can be attributed to both strong electron–electron correlations and the inverted gap band transitions due to the presence of 1T′‐phase WSe_2_. The collective observation shows the pivotal role that electron–electron correlation plays and its effects on the energy bandgap in 2D‐TMD systems.

**Figure 5:**
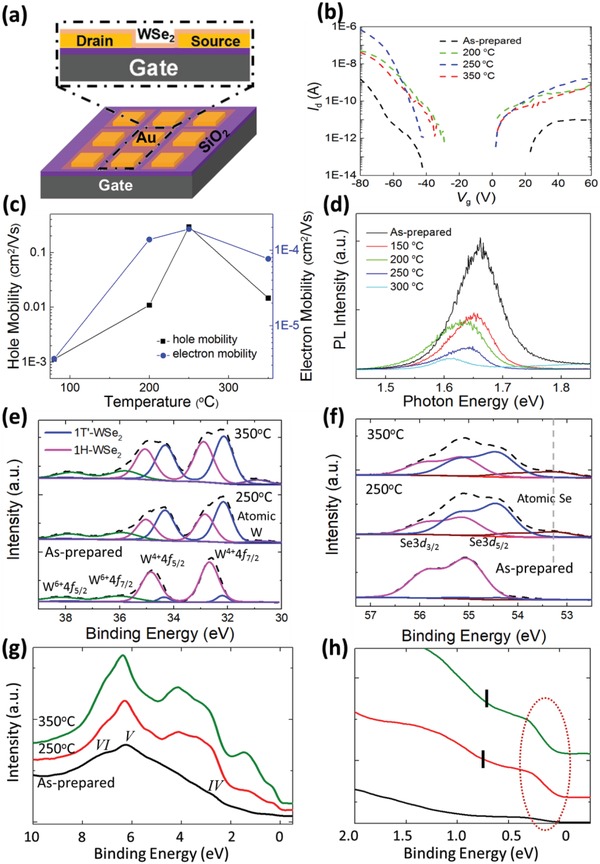
Transport, photoluminescence, and photoemission measurements of WSe_2_/Au at various annealing temperatures. a) Device structure schematic of a WSe_2_ field‐effect transistor. b) *I*_d_–*V*_g_ of the WSe_2_ device as functions of annealing temperature. c) Hole and electron mobility at different gate voltages of the device as functions of annealing temperature. d) Annealing temperature dependence of photoluminescence spectra. e,f) Synchrotron‐based X‐ray photoemission spectra of W4f, W5p3/2, and Se3d core‐level peak regions for WSe_2_ on Au film annealed at various temperatures. These spectra can be well fitted by two components with constant peak positions and full width at half maximum (FWHM). g) Valence band and h) narrow valence band for WSe_2_ on Au film annealed at various temperatures. The Fermi surface feature is indicated by the dashed red circle while the mid‐gap feature positions denoted by the black vertical markings (see the Experimental Section for details).

A new set of WSe_2_/Au devices is also fabricated (**Figure** **5**a) to study the annealing temperature‐dependent transport properties as shown in Figure 5b,c. The transport properties of the device are also very sensitive to the annealing temperature. Figure 5b shows the extracted field‐effect current, *I*_d_, as a function of the back gate voltage, *V*_g_. WSe_2_ shows ambipolar electrical behavior including both p‐ and n‐type conductivity, which is consistent with previous studies.^42^, ^43^ However, the saturated currents at positive *V*_g_ values are much lower than those at negative *V*_g_. The transport data indicate that our device behave like a hole‐doped system. Figure 5c shows the calculated field‐effect electron and hole mobilities. Rising trends in electron and hole mobilities indicate that the device performance is optimized with a reduction in device contact resistance by annealing the sample to 250 °C. The electron mobility is significantly lower than the hole mobility at each annealing temperature. The device mobility is also lower as compared to other mechanically exfoliated FET^5^ due to defects introduced during the CVD growth and transfer processes along with the presence of different Schottky barriers.

Photoluminescence measurements are further conducted on the monolayer‐WSe_2_/Au sample before and after annealing with the changes displayed in Figure 5d. For the as‐prepared sample, excitonic peak A located at ≈1.65 eV is attributed to the direct bandgap photoluminescence from the K‐point.^44^ By annealing the monolayer‐WSe_2_/Au sample at elevated temperatures, peak A is redshifted, broadened, and its intensity reduced. Similar PL changes observed in previous phase transition studies confirm the 1H‐1T′ phase transformation of monolayer‐WSe_2_.^25^, ^45^

Figure 5e,f displays the evolution of W4f and Se3d core‐level spectra, respectively, of the monolayer‐WSe_2_/Au sample before and after annealing at the elevated temperatures of 250 and 350 °C. The W4f spectrum for the as‐prepared WSe_2_/Au (Figure 5e) shows the W^4+^ 4f_7/2_ (≈32.68 eV) and 4f_5/2_ (≈34.84 eV) components of the 1H‐WSe_2_ spin–orbit doublet.^46^, ^47^ There are also the broad doublet peaks on the higher binding energy side at ≈36.0 and ≈38.2 eV ascribed to the surface W^6+^ ions.^46^ The presence of the W^6+^ ions indicates that the CVD‐grown WSe_2_ is slightly hole‐doped. In Figure 5f, the prominent peaks of the Se 3p_5/2_‐3d_3/2_ doublet for the as‐prepared monolayer‐WSe_2_ in 1H‐phase are located at ≈55.1 and ≈55.9 eV, respectively, consistent with previously reported values in ref. 47. After annealing the WSe_2_/Au sample at the respective temperatures, new doublets appear at lower binding energy similar to the trends of monolayer‐MoS_2_/Cu in both the W4f and Se3d core‐level spectra (Figure 5e,f).

The PES spectra for WSe_2_/Au are also peak‐fitted by two components with constant peak positions and full‐width‐half‐maximum (FWHM). These new peak features are found to be maximized at 250 °C, similar to MoS_2_ on Au as reported previously.^25^ By fitting of these newly formed features in the W4f and Se3d spectra region, the formation of an additional doublet at ≈0.68 eV for the W4f and ≈0.70 eV for the Se3d spectra below that of the respective core‐level doublets in 1H‐phase is revealed. The simultaneous appearance of these core‐level features upon high‐temperature annealing corresponds to the formation of 1T′‐WSe_2_ as previously reported.^48^ These collective trends are similar to those of the MoS_2_/Au^25^ and MoS_2_/Cu systems where simultaneous appearances of new doublets in both W and Se core‐levels at lower binding energies affirm the formation of the new quasi‐metallic 1T′‐WSe_2_ structure.

As the annealing temperature increases further to 350 °C, a decrease in 1T′‐phase doublets of both the W and Se core‐level spectra is observed. Figure 5e shows a growing broad peak at the binding energy of ≈30.76 eV, which indicates the partial decomposition of WSe_2_ at high annealing temperatures resulting in the formation of metallic W.^49^ This corresponds with the rise in the broad peak at ≈53.25 eV after annealing at 350 °C (Figure 5f), which is attributed to the presence of elemental Se.^50^ The extended range of the Se3d core‐level spectra to ≈60 eV before and after annealing process (Figure S8, Supporting Information) further verifies the absence of oxidized Se impurities in WSe_2_ after annealing.

To provide further understanding on how the 1H‐1T′ phase transition affects the electronic properties of monolayer‐WSe_2_, we analyze the electronic structures near the Fermi surface of monolayer‐WSe_2_/Au and are displayed in Figure 5g,h. A Fermi surface feature is already noticeable in the as‐prepared monolayer‐WSe_2_/Au sample prior to the annealing process as seen in the valence band spectrum. This suggests that a considerable percentage of 1T′‐phase WSe_2_ is already present in the as‐prepared sample (proportion of 1T′‐phase TMD to be further discussed). In the valence band spectrum of the as‐prepared WSe_2_/Au sample, the broad feature in the binding energy region of ≈8.2 eV with center at ≈6.1 eV denoted as peak V is an overlap of hybridization between W5d and Se4p orbitals.^51^, ^52^ The shoulder labeled as peak IV at ≈3 eV is attributed to the fully occupied $W5d_{z^{2}}$ valence bands and the broad shoulder labeled as peak VI at the ≈7 eV W5d‐Se4p hybridization at the Γ symmetry point.^51^, ^52^

By annealing the sample at 250 and 350 °C, there is a growth in the Fermi surface feature along with the distinct formation of the mid‐gap feature at ≈0.8 eV below peak IV as prominently observed in the narrow valence band spectra (indicated by vertical black markings in Figure 5h, details in the Experimental Section). Similar to monolayer‐MoS_2_ on Au, this mid‐gap feature is attributed to the band inversion of 1T′‐WSe_2_ due to symmetry breaking during the 1H‐1T′ phase transition.^8^

### 2.4 Comparison of Phase Transition Yield

**Figure 6:**
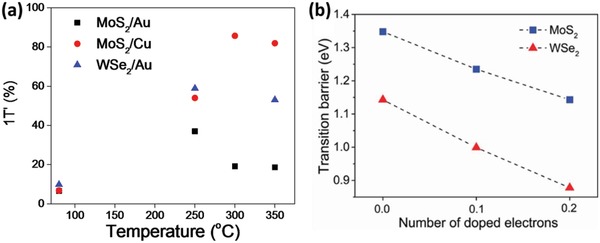
1T′‐phase proportion and phase transition barriers. a) Extracted percentage yield of 1T′‐phase components of Mo3d_5/2_ and W4f_7/2_ peaks as functions of annealing temperature from XPS core‐level spectra. The data of MoS_2_/Au are extracted from ref. 25. b) The phase transition barrier of the MoS_2_ and WSe_2_ monolayers from 1H‐phase to 1T′‐phase with and without electron doping.

With the confirmation of the 1H‐1T′ phase transition dynamics of both the MoS_2_/Cu and WSe_2_/Au systems, we proceed to analyze the percentage yield in 1T′‐phase of these two systems and compare it with our previous study on MoS_2_/Au.^25^ **Figure** **6**a displays the percentage (Table S1, Supporting Information) of 1T′‐MoS_2_ components of Mo3d_5/2_ peaks for the MoS_2_/Cu and MoS_2_/Au^25^ samples and that of 1T′‐WSe_2_ component of W4f_7/2_ (WSe_2_/Au) as functions of annealing temperature. The proportion of the new S2p_3/2_ peak (Se3d_5/2_ peak) is not taken for the percentage yield analysis exactly due to the presence of S (Se) vacancies in monolayer‐MoS_2_ (WSe_2_) during the growth and transfer processes. From a percentage of merely ≈7% (MoS_2_/Cu and MoS_2_/Au) and ≈10% (WSe_2_/Au) 1T′‐phase component before annealing, a maximum yield of ≈37% in 1T′‐MoS_2_/Au was previously observed at the annealing temperature range of 200–250 °C. Interestingly, this work shows a significant increase in yield for 1T′‐MoS_2_/Cu to ≈85.7% at the optimum annealing temperature of 300 °C, and ≈58.8% for 1T′‐WSe_2_/Au at 250 °C.

The significant increase in percentage yield of 1T′‐phase 2D‐TMDs via this straightforward annealing‐based technique holds important implications. While MoS_2_/Cu records an optimum percentage yield at 300 °C, both MoS_2_/Au and WSe_2_/Au have their 1T′‐phase yields optimized in the annealing temperature range of ≈250 °C. This indicates the conditions of optimal surface chemical reactivity of the metallic substrate. This is consistent with previous annealing‐temperature‐dependent studies involving several metal substrates which reported a reduction in the contact resistance between multilayer MoS_2_ films and Au after annealing at 250 °C, while the contact resistance in systems such as MoS_2_ on titanium being minimized after annealing at 300 °C.^53^

A first‐principles study (details in the Experimental Section) is conducted to compare the phase transition barriers of WSe_2_ and MoS_2_ between the respective 1H and 1T′ phases at different electron doping levels as displayed in Figure 6b and Figure S1 (Supporting Information). Noticeably, the 1H‐1T′ transition energy barrier for monolayer‐WSe_2_ is ≈0.2 eV lower than that of monolayer‐MoS_2_. This energy difference may be attributed to the different bond strengths between the 1H‐phase monolayer‐MoS_2_ and WSe_2_. Structural phase transition is harder to realize for the system with stronger covalent bonds. This is confirmed by the calculated bond energy where the Mo—S bond energy of 1H‐phase monolayer‐MoS_2_ system is ≈0.3 eV larger than that of the W—Se bond in the 1H‐phase monolayer‐WSe_2_ system. These phase transition barriers can be further tuned via electron doping, such that the phase transition barrier is reduced significantly for both monolayer‐MoS_2_ and WSe_2_. Results from this first‐principles study provide better physical insight to the experimentally observed phase transition of different 2D‐TMDs. Thus, both the choice of metallic substrates and 2D‐TMDs can enhance the 1H‐1T′ phase transition yield significantly by different mechanisms: increasing interfacial hybridizations or reducing the intrinsic transition‐energy barrier. This work suggests that increasing the interfacial hybridizations can be more effective than reducing the 1H‐1T′ phase transition energy barrier.

## 3 Conclusion

In conclusion, the collective general trends of this annealing‐based phase transition study of 2D‐TMDs on metallic substrates have shown that the choice of metallic substrate with high chemical reactivity and the option of 2D‐TMD with low intrinsic 1H‐1T′ energy barriers can significantly dictate the yield of 1T′‐phase 2D‐TMD. Moreover, our study demonstrates the unprecedented quality of the 2D‐TMD/metallic substrate interface at the atomic length scale, which in turn can be a major step forward toward general interest. This work opens up the field of interfacial studies of 2D‐TMDs and metallic substrates for further research. Our comprehensive experimental and theoretical study provides a deeper insight and substantial understanding to the underlying interfacial dynamics in the monolayer‐TMD and metallic substrate system. Insights into the underlying mechanism and the ability to effectively manipulate the experimental parameters to optimize phase transition yield promising exciting future developments in 2D‐TMD‐based device applications.

## 4 Experimental Section

*Sample Preparation*: Spectroscopic ellipsometry and photoemission spectroscopy measurements require high‐quality and large‐area MoS_2_ (WSe_2_) monolayers. MoS_2_ (WSe_2_) atomic layers were synthesized on sapphire surface by CVD method using MoO_3_ (WO_3_) and S (Se) powders as the reactants.^25^, ^44^, ^54^ Both the CVD‐grown MoS_2_ and WSe_2_ monolayer sheets were chemically transferred onto their respective metallic foil (MoS_2_ on Cu (111)–MoS_2_/Cu and WSe_2_ on Au (111)–WSe_2_/Au) using polymethyl methacrylate (PMMA). In the case of the Au foil for the monolayer‐WSe_2_ sample, a 200 nm gold film was sputter‐coated on SiO_2_/Si substrate. The TEM measurements were performed in a Tecnai G2 F30 transmission electron microscope with an acceleration voltage of 300 kV. During the HRTEM characterization of WSe_2_/Au, the electrons were strongly absorbed and blocked by the noble metal substrate. Hence, the Au film thickness was reduced to 5–10 nm. However, in the case of MoS_2_ on 5–10 nm Cu substrate, the thin substrate was easily oxidized. This strongly affects the results. Hence, no good HRTEM result for the MoS_2_/Cu sample was obtained. They were then annealed at 80 °C to enhance the contact between the film and substrate as well as to eliminate any existing residues (as‐prepared samples). Raman and photoluminescence spectra for the CVD‐grown monolayer‐WSe_2_ are shown in Figure S7 (Supporting Information). In comparison with the recent report of monolayer‐WSe_2_ on sapphire,^44^ the spectra are similar, thus indicating that the sample is of high monolayer quality. An MoS_2_ (WSe_2_) FET was fabricated for electrical transport, photoluminescence, and Raman spectroscopic measurements. The 20/60 nm Ti/Cu (Ti/Au) electrode array on SiO_2_/Si substrate was evaporated when the SiO_2_/Si substrate was covered by a Cu grid. The MoS_2_ (WSe_2_) film was transferred onto the electrodes via the PMMA‐assisted method, to create the MoS_2_/Cu (WSe_2_/Au) interfaces. The two Ti/Cu (Ti/Au) electrodes and SiO_2_/Si substrate formed the source, drain, and gate electrodes, respectively. The bias voltage between drain and source is 1 V. The device was annealed at various temperatures for 15 min in vacuum, and all the measurements were performed after the device was cooled to room temperature.

*Synchrotron‐Based High‐Resolution PES Measurements*: To provide a detailed study into the unique properties of 2D‐TMDs, X‐ray photoemission spectroscopy (XPS) serves as an effective and powerful characterization technique to distinguish between different exotic phases and species.^19^, ^25^, ^31^, ^45^ By analyzing the proportion of the core‐level peaks, the percentage yield of the TMDs in their respective phases can also be conveniently monitored and accurately elucidated.^25^, ^31^

The monolayer MoS_2_/Cu and WSe_2_/Au samples were annealed at various temperature in ultrahigh vacuum chamber with a base pressure of 1 × 10^−10^ mbar at the surface, interface, and nanostructure science (SINS) beamline of the Singapore Synchrotron Light Source for PES measurements. The MoS_2_/Cu sample was annealed using the filament at the following temperatures for 15 min each: 200, 250, 300, 350, 400, 450, 500, and 550 °C. Whereas, the WSe_2_/Au sample was annealed at 250 and 350 °C before cooling down to room temperature for PES measurements. After which, the samples were rapidly cooled back to room temperature by turning off the heating filament current. This is followed by the synchrotron‐based PES measurements which were performed immediately after the sample was cooled to room temperature. The photon energy of 365 eV was used to probe the Mo3d, S2p and 60 eV for the valence band spectra of the MoS_2_/Cu system. Similar photon energy of 365 eV was used for W4f, and Se3d and 60 eV for the valence band spectra of the WSe_2_/Au system. All PES spectra were collected at normal emission using a VG Scienta R4000 analyzer and normalized by a photon current. The respective photon energies were calibrated using the Au4f_7/2_ core‐level peak at 84.0 eV of a sputter‐cleaned gold foil in electrical contact with the sample. The binding energy is taken with reference to the Fermi level of gold foil. Least‐square peak fitting analyses were performed using Voigt photoemission profiles with constant Lorentzian (15%) and Gaussian (85%) line shapes. For Mo3d and S2p spectra fitting, splitting difference of ≈3.15 eV with branching ratio of 3 (Mo3d_5/2_):2 (Mo3d_3/2_) and ≈1.18 eV with branching ratio of 2 (S2p_3/2_):1 (S2p_1/2_) were used, respectively. As for the W4f and Se3d spectra fitting, split ratios of ≈2.16 eV were used with a branching ratio of 4 (W4f_7/2_):3 (W4f_5/2_) and ≈0.8 eV with branching ratio of 3 (Se3d_5/2_):2 (Se3d_3/2_), respectively.

*High‐Resolution Spectroscopic Ellipsometry Measurements*: A J. A. Woollam Co., Inc. spectroscopic ellipsometer with photon energy of 0.4–3.2 eV was used to measure the ellipsometry parameters Ψ (the ratio between the amplitude of p‐ and s‐polarized reflected light) and Δ (the phase difference between p‐ and s‐polarized reflected light). The refractive index *n* and extinction coefficient *k* of WSe_2_ were extracted from the parameters Ψ and Δ utilizing an air/WSe_2_/substrate multilayer model, where the WSe_2_ film comprises an average homogeneous and uniform medium. Hence, the optical data of the top WSe_2_ layer were derived by eliminating the optical signals from the bottom substrate (with optical data measured separately by spectroscopic ellipsometry in the same condition).

*First‐Principles Calculations*: The first‐principles calculations were performed within the framework of DFT using Vienna ab Initio Simulation Package (VASP5.4.4).^55^, ^56^ Projector augmented wave (PAW) potentials were selected to account for the interactions between electrons and ions.^57^ The generalized gradient approximation (GGA) with Perdew–Burke–Ernzerhof (PBE) format^58^ was used for the electron exchange‐correlation function. The kinetic cutoff energy of plane‐wave basis to expand electronic wave functions was set to 500 eV. The first Brillouin zone of monolayer 1H‐MoS_2_ (WSe_2_), 1T'‐MoS_2_(WSe_2_), graphene, bulk Au(Ag, Cu), MoS_2_/Au interface, MoS_2_/Ag interface, MoS_2_/Cu interface, and MoS_2_/graphene interface was sampled by Γ‐centered 12 × 12 × 1, 8 × 12 × 1, 16 × 16 × 1, 10 × 10 × 10, 2 × 2 × 1, 2 × 2 × 1, 3 × 3 × 1, and 3 × 3 × 1 K‐point meshes, respectively. The geometries of all structures were optimized until the force on each atom was smaller than 0.01 eV Ǻ^−1^. The supercell size of the MoS_2_, Au (111), Ag (111), Cu (111), and graphene was used to construct the heterostructures as shown in Table 1. Four atomic layers were used for the Au (111), Ag (111), and Cu (111) substrates. During the structural optimization, the bottom two layers of metal atoms were fixed while other atoms were fully relaxed. To minimize the artificial Coulomb interactions of the monolayer TMDs, graphene, and interface structures due to the periodic condition, a vacuum region larger than 15 Å was applied to monolayer structures, and for the MoS_2_/metals heterostructures, a vacuum layer larger than 10 Å was used. The van der Waals (vdW) effects on MoS_2_/Au(111), MoS_2_/Ag(111), MoS_2_/Cu (111), and MoS_2_/graphene were considered by using Crimme's DFT‐D3 method.^59^

The transition barriers from 1H to 1T′ phase of MoS_2_ (WSe_2_) were calculated by using c‐NEB method.^60^ Five images were inserted between the 1H (rectangular supercell) and 1T′ phases and the force was optimized smaller than 0.01 eV Ǻ^−1^. The bonding energies of W—Se and Mo—S bonds in 1H‐WSe_2_ and 1H‐MoS_2_ were calculated by using the following formula

(1) $$E_{\mathrm{bonding}}\;=\left(E_{\mathrm{supercell}}-E_{D}-\mu \right)/N_{\mathrm{bonds}}$$

in which *E*_supercell_ is the total energy of the perfect 1H‐MoS_2_(WSe_2_) supercell, *E*_D_ is the total energy of the supercell in which a S or Se atom was removed to break the Mo—S or W—Se bonds, and μ is the chemical potential of the S or Se atom. In this study, the energy of S_2_ molecule and trigonal phase of Se bulk^61^ was selected as the reference. *N*_bonds_ is the coordination number of Mo—S (W—Se) bonds. 3 × 3 × 1 supercell was used to calculate the bonding energy. The energy curve along the structure transition path from monolayer 1H‐MoS_2_ to 1T′‐MoS_2_ and 1H‐WSe_2_ to 1T′‐WSe_2_, calculated by c‐NEB, are shown in Figures S1 and S2 (Supporting Information).

The lattice constants of 1H and 1T′‐MoS_2_ primitive cell are calculated to be 3.18 Å, and *a* = 5.73 and *b* = 3.17 Å, respectively. While for WSe_2_, the corresponding lattice constants are optimized to be 3.32, and *a* = 5.96 and *b* = 3.31 Å, respectively. All these results are in good agreement with previous studies.^62^, ^63^, ^64^ The (√3 × 1) 1H‐MoS_2_ and WSe_2_ supercells were used for the calculations of the transition barriers as shown in Figure S3 (Supporting Information), where the 1T′ phases were stretched slightly to match the lattice constants of the corresponding 1H phases.

To better deduce the changes in the electronic structure at the interface between 2D‐TMD and metallic substrate induced through the annealing procedure, the ab initio molecular dynamics simulations were performed with a time step of 1 fs, total simulation time of 3.5 ps, canonical ensemble (*NVT*), and a Nosé heat bath. Figure S4 (Supporting Information) shows the temperature evolution of the 1H‐MoS_2_/Cu during the molecular dynamics simulations annealing at 550 K.

*Influence of Bond Strength in Phase Transition*: The bond strength should play an important role to the onset of 1H‐1T′ structural phase transition. According to the computational results, the lattice structures of 1H‐MoS_2_ and 1T'‐MoS_2_ are significantly different. In 1H‐MoS_2_, all the Mo—S bond length and Mo—S—Mo bond angle stand consistently at 4.21 Å and 82.50°, respectively. The sliding and distortion of Mo and S atoms during the phase transition process can lead to various bond lengths and bond angles. The Mo—S bond lengths in 1T'‐MoS_2_ range between 2.39 and 2.51 Å. The bond with weaker strength is expected to be distorted easily.

*Lattice Distortion Induced by the Thermal Effect in Molecular Dynamic Simulations*: In the molecular dynamics simulations which depict the high‐temperature annealing process, the length of Mo—S bond in MoS_2_/Cu ranges from 2.40 to 2.46 Å in optimized ground state (Figure S5a, Supporting Information). Whereas, it is ranged between 2.30 and 2.63 Å after the molecular dynamics simulations (Figure S5b, Supporting Information). The larger range in bond length after the molecular dynamics simulations indicates larger lattice distortion and weaker bond strength. This increases the possibility of phase transition induced by higher annealing temperature.

*Estimation of Energy Positions of Features from Valence Band Spectra*: The broadening of the valence band photoemission spectra can be caused by some sample features, namely, surface roughness, sample defects, impurities, and vacancies. In situations when the peak is broad, defining the energy positions of features to be located at the middle of the edge would undermine its accuracy. Hence, the estimate energy positions of mid‐gap and Fermi surface features (0 eV) in Figures 2d and 3d are derived by intersecting the extrapolated leading edge with the background baseline. Prior to measurement, the photon energy was calibrated using Fermi‐edge position of a sputter‐cleaned gold foil in electrical contact with the sample. This calibration was also derived by intersecting the extrapolated leading edge with the background baseline (0 eV), instead of the middle of the Fermi edge.

## Conflict of Interest

The authors declare no conflict of interest.

## Supporting information

Supplementary
