# Supplementary material for: Unraveling High‐Yield Phase‐Transition Dynamics in Transition Metal Dichalcogenides on Metallic Substrates
Source: Adv Sci (Weinh). 2019 Jan 25;6(7):1802093. doi: 10.1002/advs.201802093 (PMC6446595; doi:10.1002/advs.201802093)
Supplement: Supplementary file 1 — Supplementary [file ADVS-6-1802093-s001.pdf]

Copyright WILEY-VCH Verlag GmbH & Co. KGaA, 69469 Weinheim, Germany, 2019.

ADVANCED SCIENCE
Open Access

Supporting Information

for *Adv. Sci.,* DOI: 10.1002/advs.201802093

Unraveling High-Yield Phase-Transition Dynamics in Transition Metal Dichalcogenides on Metallic Substrates

Xinmao Yin, Chi Sin Tang, Di Wu, Weilong Kong, Changjian Li, Qixing Wang, Liang Cao, Ming Yang, Yung-Huang Chang, Dianyu Qi, Fangping Ouyang, Stephen J. Pennycook, Yuan Ping Feng, Mark B. H. Breese, Shi Jie Wang, Wenjing Zhang, Andrivo Rusydi,* and Andrew T. S. Wee**

Supporting Information

Unravelling high-yield phase-transition dynamics in transition metal dichalcogenides on metallic substrates

Xinmao Yin, Chi Sin Tang, Di Wu, Weilong Kong, Changjian Li, Qixing Wang, Liang Cao, Ming Yang, Di Wu, Yung-Huang Chang, Dianyu Qi, Stephen John Pennycook, Yuan Ping Feng, Mark B. H. Breese, Shi Jie Wang, Wenjing Zhang*, Andrivo Rusydi*, Andrew T. S. Wee*

Supporting Figures

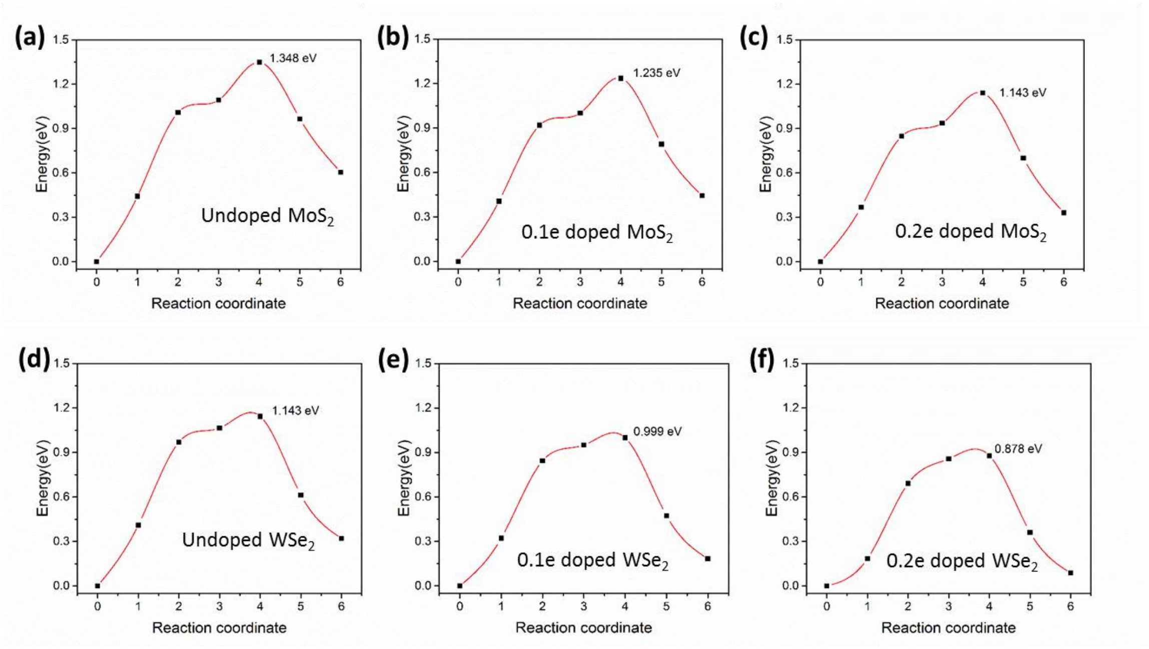

Figure S1. Energy curve for 7 steps of the process of phase transition in 2D-TMDs. The energy curve along the structure transition path from monolayer a-c) 1H-MoS_2 to 1T’-

MoS_2, and from monolayer d-f) 1H-WSe_2 to 1T'-WSe_2, calculated by c-Nudged Elastic Band method, where different images are noted by the reaction coordinate.

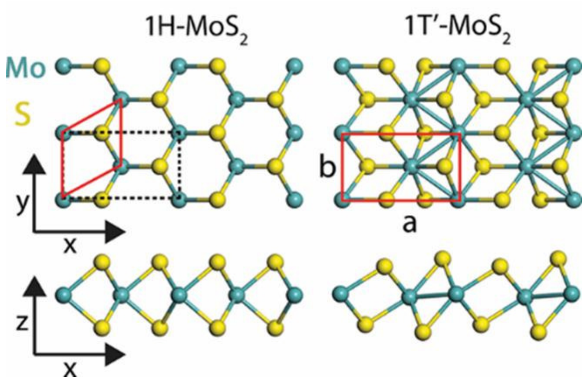

Figure S2. Atomistic structures of different phases of MoS_2. Atomistic structures of 1H and 1T'-phase MoS_2, in which the primitive cells are denoted as red solid lines, respectively.

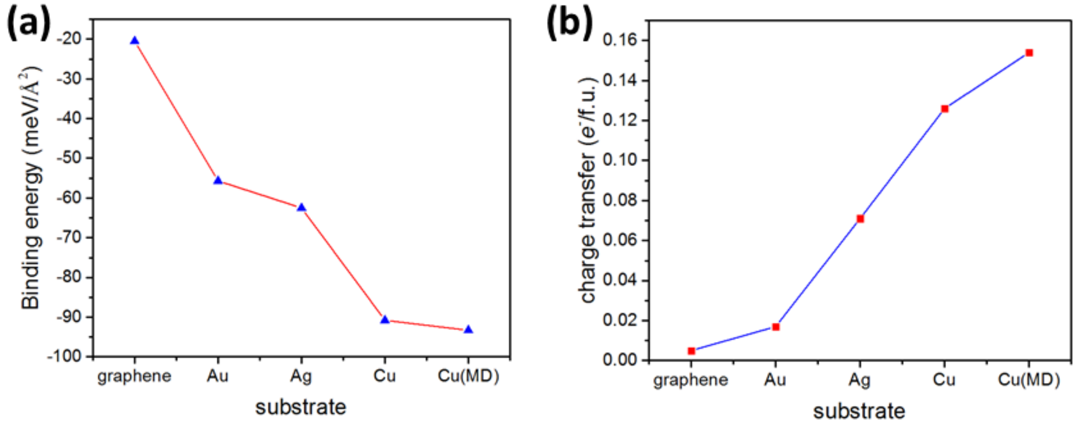

Figure S3. Calculated interfacial hybridizations between MoS_2 and metallic substrates. a) Binding energy of 1H-MoS_2 with graphene, Au, Ag, and Cu. b) Charge transfer from different substrates to 1H-MoS_2. Cu(MD) denotes the MoS_2/Cu after molecular dynamics simulations.

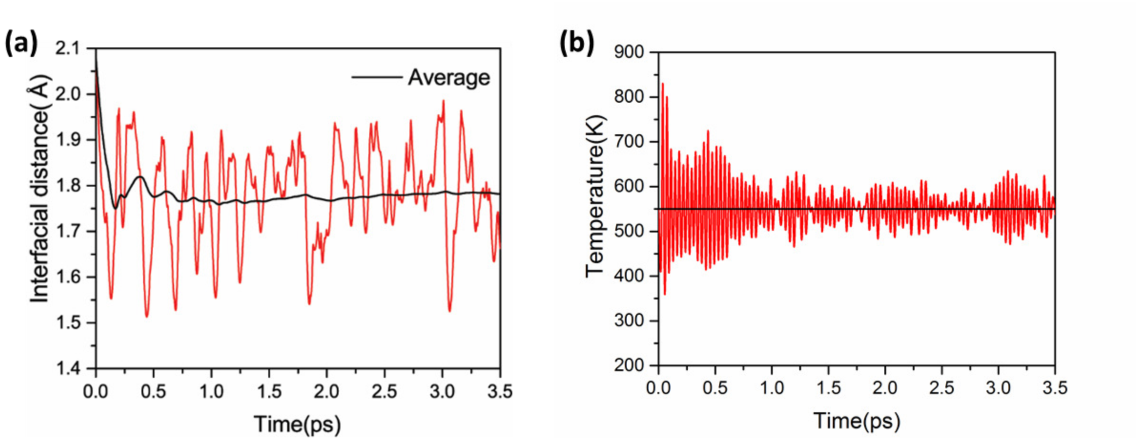

Figure S4. Molecular dynamics simulations after annealing of MoS_2 on Cu. a,b) The interfacial distance variations versus the time and the temperature evolution of the 1H-MoS_2/Cu during molecular dynamics simulations after annealing at 550K. The red line in (a) is the variation of the interfacial distance between monolayer 1H-MoS_2 and Cu substrate, the black line in (a) is the averaged interfacial distance which averages all the distances gained in previous time.

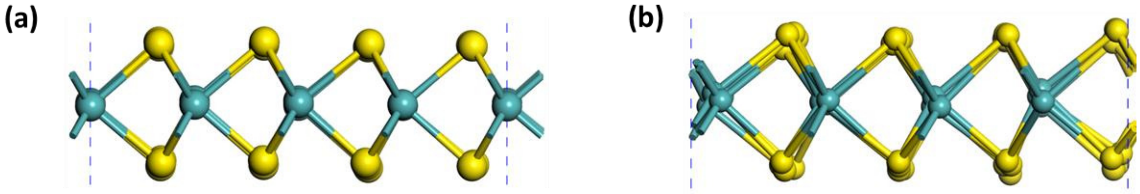

Figure S5. The structure change of MoS_2 induced by the thermal effect in molecular dynamic simulations. a,b) The distortion of the bonds (a) at ground state, and (b) after molecular dynamics simulations with annealing temperature of 550K in monolayer-MoS_2. Details in Experimental Section.

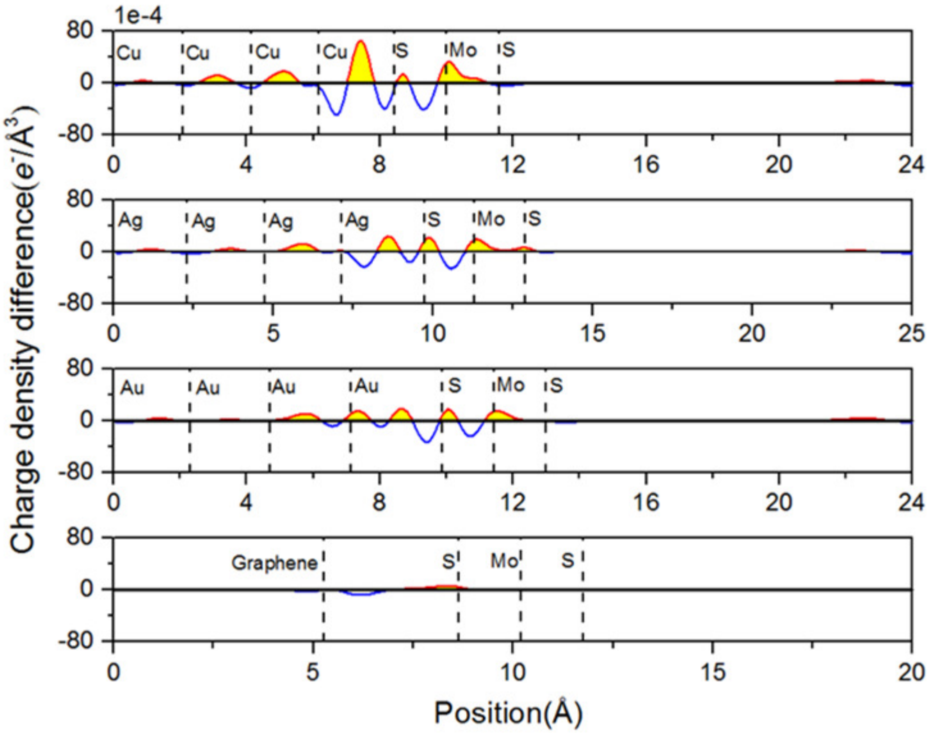

Figure S6. Calculated charge density difference of MoS_2 on metallic substrates. Averaged charge density difference along the Z direction of 1H-MoS_2 on graphene, Au, Ag, Cu. The charge accumulation between bottom S atomic layers and the top substrate layers indicates the hybridization of MoS_2 with the substrate. The charge accumulation on Mo atomic layers and charge depletion between Mo atomic layer and interfacial S atomic layer indicate the hybridization between Mo and S atomic layers would be weakened thus decreased the Mo-S bond strength.

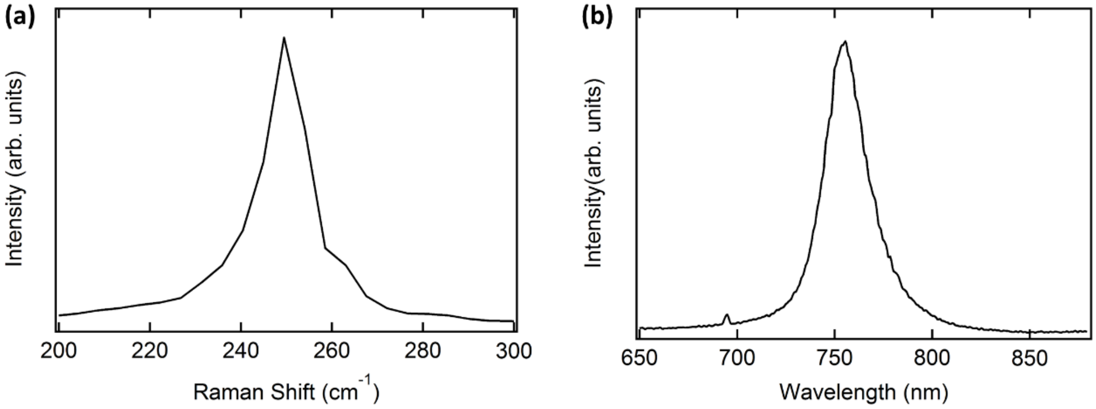

Figure S7. Raman and Photoluminescence spectra for the CVD monolayer-WSe_2 on sapphire. a) Raman spectra, where the two characteristic peaks for monolayer-WSe_2 at ~250 cm^{-1} (E^1_{2g} mode), and ~261 cm^{-1} (A_{1g} mode). b) Photoluminescence spectra, a strong emission at ~755 nm corresponding to the A excitonic absorption for monolayer-WSe_2.

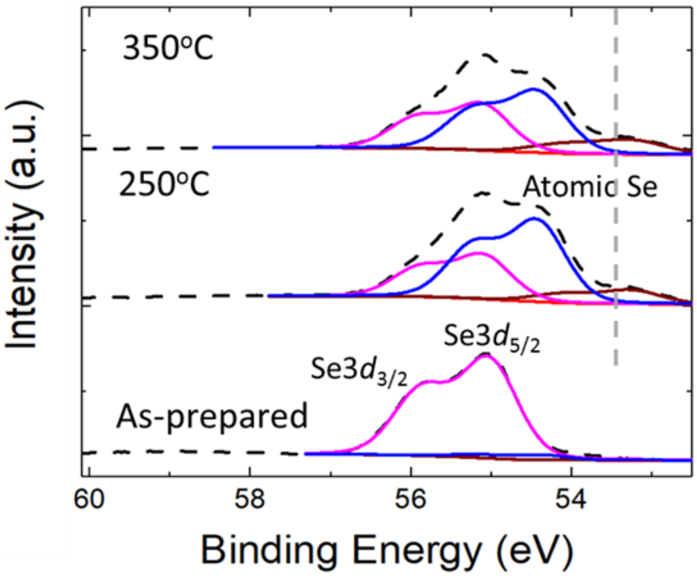

Figure S8. Synchrotron based X-ray photoemission spectra of Se 3d core level peak regions of WSe2 on Au film annealed at various temperatures.

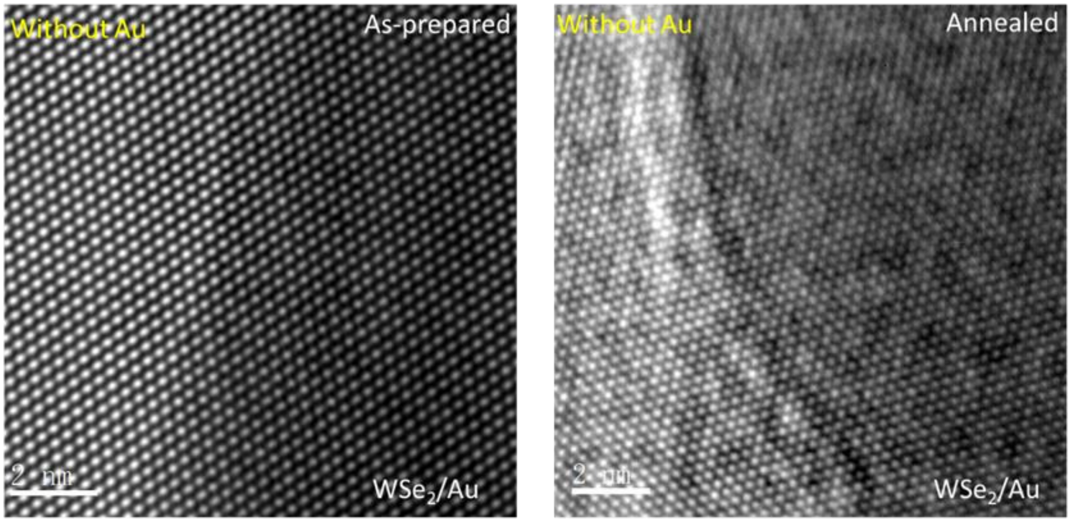

Figure S9. HRTEM images of WSe2/Au before (left) and after (right) annealing at 250 ^{o}C.

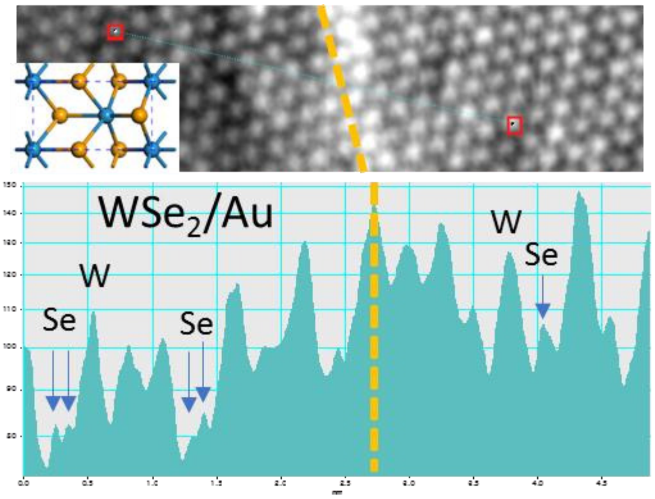

Figure S10. Intensity profiles along the blue lines indicated in above HRTEM images of annealed (at 250°C) samples. The dark region shows the region where monolayer-WSe_2 lies above the Au substrate (WSe_2/Au), while the other is the region where monolayer-

WSe_2 is suspended in vacuum. The yellow lines show the boundary between these two regions.

Supporting Tables

**Annealing Temperature (^oC)**	**$Mo3d_{5/2}$ in 1T'-phase (%)**
As-prepared	7.0
250	54.0
300	85.7
350	81.9

Table S1. Yield in 1T'-phase MoS_2/Cu after annealing. Percentage yield in 1T'-phase MoS_2/Cu as represented by the $Mo3d_{5/2}$ proportion upon annealing at the respective temperatures.

**Annealing Temperature (^oC)**	**$W4f_{7/2}$ in 1T'-phase (%)**
As-prepared	10.0
250	58.8

Table S2. Yield in 1T'-phase WSe_2/Au after annealing. Percentage yield in 1T'-phase WSe_2/Au as represented by the $W4f_{7/2}$ proportion upon annealing at the respective temperatures.
